# Enhancement of the productivity of the potent bacteriocin avicin A and improvement of its stability using nanotechnology approaches

**DOI:** 10.1038/s41598-017-10157-9

**Published:** 2017-09-06

**Authors:** Hazem A. Fahim, Waleed M. A. El Rouby, Ahmed O. El-Gendy, Ahmed S. Khairalla, Ibrahim A. Naguib, Ahmed A. Farghali

**Affiliations:** 1 Biotechnology and Life Sciences Department, Faculty of Postgraduate Studies for Advanced Sciences (PSAS), Beni-Suef University, Beni-Suef, Egypt; 2 Materials Science and Nanotechnology Department, Faculty of Postgraduate Studies for Advanced Sciences (PSAS), Beni-Suef University, Beni-Suef, Egypt; 3 Microbiology and Immunology Department, Faculty of Pharmacy, Beni-Suef University, Beni-Suef, Egypt; 4 Pharmaceutical Analytical Chemistry Department, Faculty of Pharmacy, Beni-Suef University, Beni-Suef, Egypt

## Abstract

Herein, enhancements of the yield and antimicrobial activity duration of the bacteriocin avicin A were accomplished using fractional factorial design (FFD) and layered double hydroxide (LDH) nanoparticles. Firstly, potential factors affecting bacteriocin production were selected for preliminary study. By a 2^5-1^ FFD, high pH was shown to have a positive effect on avicin A yield, while temperature and duration of incubation, as well as peptone nitrogen sources all had negative effects. The highest bacteriocin production and activity (2560 BU/ml) were observed after 30 h of incubation at 30 °C, with pH adjustment at 7, and in the presence of 2 g mannitol as carbon source and 2.2 g peptone as nitrogen source. Secondly, avicin A nanocomposites with different LDH precursors were tested. Only avicin A-ZnAl-CO_3_ LDH demonstrated a potent antimicrobial activity against *Lactobacillus sakei* LMGT 2313 that lasted for at least 24 days, as compared to the values of 6 and 15 days observed with the free avicin A that has been stored at room temperature and at 4 °C, respectively. In conclusion, avicin A production and stability can be improved by manipulating the growth conditions and media composition, together with conjugation to LDHs.

## Introduction

Bacteriocins are a miscellaneous group of proteinaceous compounds, produced by diverse bacteria, which usually demonstrate antibacterial activity against species closely related to the bacteriocinogenic strain^1^. Generally, bacteriocins have been used as biopreservatives in the food industry and as antibacterial agents in the biomedical sector^2, 3^. These two applications are accomplished either by the direct use of bacteriocin in a relatively pure form, or through the incorporation of a bacteriocin-producer strain (as a probiotic)^2^.

Despite their promising applications, the commercial availability of bacteriocins is quite limited, due to challenges attributed to the proteinaceous nature of these compounds^4^. These challenges include: (i) the high costs for large scale production^5^; (ii) the low yield of the product, as a result of depending on complex media and ineffective purification methods^6, 7^; and (iii) the loss of activity due to proteolytic degradation and/or unfavourable interactions with other food components^5, 8^. Therefore, these challenges have to be met to enable a more efficient use of these compounds. Among the possible approaches to overcome the low yield and the high production costs of bacteriocins is to examine the variables affecting bacteriocin production. In this regard, different studies have examined the effects of various media composition and culture conditions on the yield of bacteriocins^9, 10^. However, most of these studies have adopted conventional methods to maximize the yield, by changing one independent variable at a time while keeping other variables fixed. In addition of being extremely time consuming and expensive, especially for a large number of variables, these methods may also result in wrong conclusions^11, 12^. Furthermore, neglecting the influence of interactions between the different factors is a major drawback of these methods. In order to save money and efforts, a fractional factorial experimental design was applied in this study to assess the direct effect of each of the significant parameters, as well as to evaluate the possible interactions between these parameter, and thus have been crucial in defining the most influential conditions for maximum production of bacteriocin^13^.

The use of nano-carrier systems including liposomes, chitosan, nanofibers, and metal nanoparticles are relatively new approaches to protect bacteriocins from degradation^4^. However, the resulting nanoformulations have been found to vary in their ability to maintain their biological activity over long storage periods^4^. Therefore, it is of great interest to determine the appropriate nanotechnological approaches for each bacteriocin individually. Layered double hydroxides (LDHs) are a family of anionic clay materials that have the ability to intercalate neutral guest molecules and/or to exchange inorganic and organic ions between hydroxide layers^14^. Given their large surface area, biocompatibility, high anion exchange capacity, and chemical stability, LDHs have gained a considerable attention as a carrier for drugs and biological compounds^15, 16^. Therefore, conjugation of bacteriocins with LDH could represent a potential solution to extend the shelf life and improve the commercial value of these antimicrobial peptides.

In a previous study, a bacteriocin-producing *Enterococcus avium* isolate was found to produce a new pediocin-like bacteriocin, named avicin A^17^. This bacteriocin has been shown to possess a potent antimicrobial activity against many species of Gram-positive bacteria, including the food-borne pathogen *Listeria monocytogenes* ^17^. Therefore, with an ultimate goal of enhancing the yield and stability of avicin A, the specific aims of the present study were to: (i) examine the effects of various pH values, incubation temperatures, incubation time, and medium components (including different types of carbon and nitrogen sources) on the yield of avicin A produced by *E. avium* HF86, and (ii) investigate the antimicrobial activity and stability of avicin A-LDH nanocomposites against Gram-positive and -negative bacteria.

## Confirmation of the Identity of the Bacteriocinogenic Isolate and Avicin A

**Figure 1:**
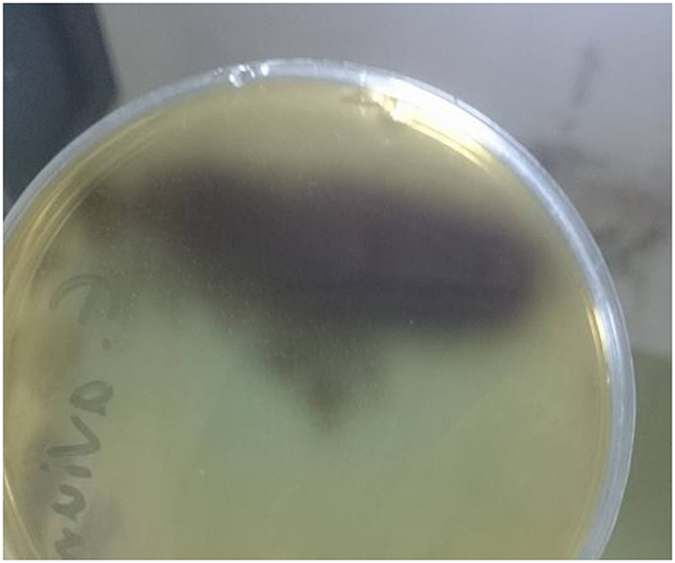
The growth of *E. avium* HF86 on bile esculin agar. The *E. avium* HF86 was tested after incubation for 24 and 48 h for esculin hydrolysis on bile esculin agar (Difco Laboratories; Detroit, USA). The formation of shiny black colonies confirms the identification of the test strain.

**Figure 2:**
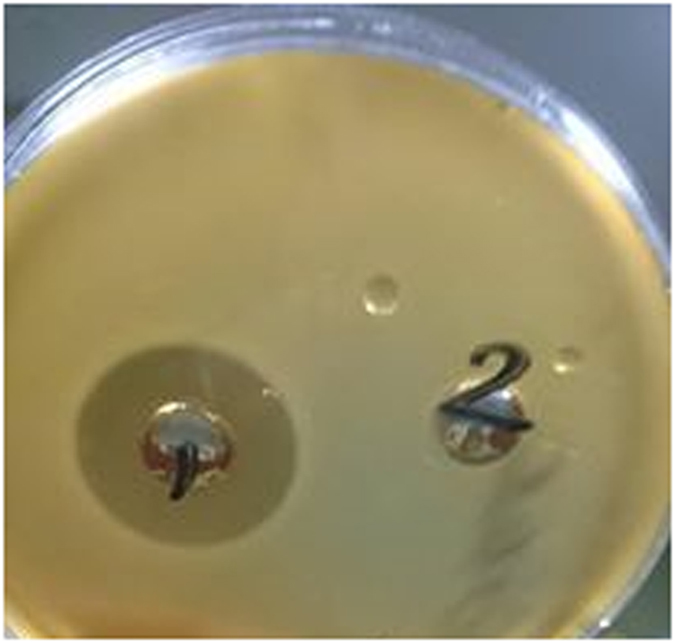
The antibacterial activity and the protein nature of avicin A. The antibacterial activity of avicin A was checked against *L. sakei* LMGT 2313, using the agar well diffusion method. While crude avicin A from the supernatant, which has been adjusted to pH 6.5, showed high antibacterial activity, as indicated by its ability to inhibit the growth of the sensitive indicator strain (well number 1), this activity was abolished completely when the supernatant was pre-treated with 1 mg/ml of proteinase K enzyme (Sigma) for 60 min at 37 °C (well number 2), indicating the protein nature of avicin A.

**Figure 3:**
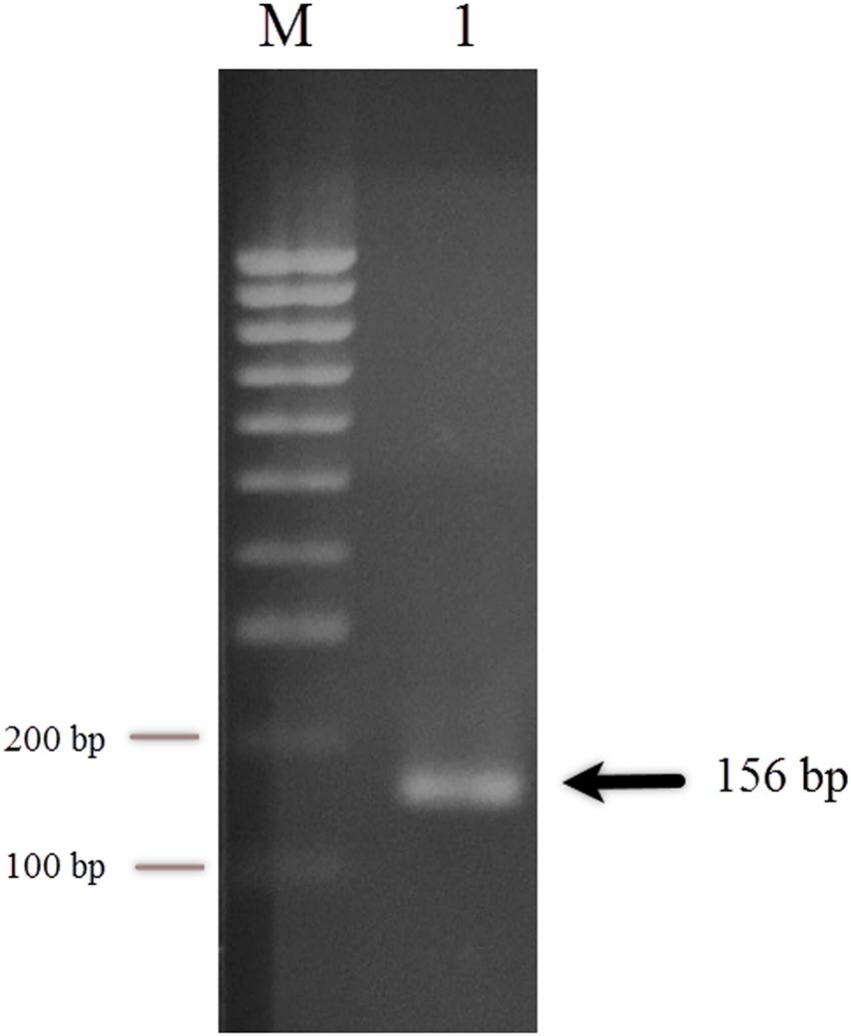
RT-PCR detection of avicin A mRNA. Total RNA (1 μg) isolated from *E. avium* HF86 cells was reverse transcribed, and the resulting cDNA was amplified by a 35-cycle PCR with the primer pairs AV-F and AV-R (Table 1). The PCR product was visualized after agarose gel electrophoresis (2%) with ethidium bromide. Lane 1 shows the reverse-transcribed PCR product of avicin A, and the arrow indicates the size of the product. The DNA molecular size marker is shown in the leftmost lane (lane M). Full-length gel is presented in Supplementary Figure S1.

*E. avium* HF86 showed shiny black colonies on bile esculin agar, as a result of esculin hydrolysis (Fig. 1). When the antimicrobial activities of 9 antibiotics were tested against *E. avium* HF86, the isolate showed sensitivity to vancomycin and gentamicin only, while it was resistant to ampicillin/sulbactam, cefotaxime, ceftriaxone, cefoperazone, ceftazidime, erythromycin, and tetracycline (data not shown). In an effort to confirm the identity of the test strain, the DNA encoding the 16S rRNA was PCR-amplified using primers listed in Table 1, sequenced, and deposited in GenBank with accession number KY921715, which when blasted to NCBI RefSeq set, it showed 99% similarity to the *E. avium* XA83 strain. In terms of bacteriocin production, the test isolate produced bacteriocin, as indicated by its ability to inhibit the growth of the sensitive indicator strain *L. sakei* LMGT 2313 (Fig. 2). The loss of bacteriocin activity after treatment with proteinase K enzyme (Fig. 2) confirmed the proteinaceous nature of the inhibitory substance. Also, the size of the reverse-transcribed PCR product (156 bp, Fig. 3) added further strong support to the identity of our bacteriocin being avicin A.

**Table 1:** PCR primers used in this study.

Target gene	Primer sequence (5′ → 3′ direction)^*^	Amplicon size (bp)	Annealing temperature (°C)	Reference
16 s (SSU) rRNA	1F: GAGTTTGATCCTGGCTCAG	1106	56	36, 37
	12R: AGGGTTGCGCTCGTTG			
Avicin A	AV-F: ACGCGAAATGAAGAATGTTG	156	55	38
	AV-R: GACTTCCAACCAGCAGCAC			

*F: forward primer; R: reverse primer.

## The Effects of Medium Composition and Growth Conditions on Bacteriocin Production

Based on the preliminary experiments: mannitol, lactose, peptone, protease peptone, initial pH 7 and 8, incubation periods of 18 and 30 hours, and incubation temperatures of 30 °C and 20 °C had a significant effect (*p* ˂ 0.05) on avicin A production. With regard to carbon source, while all the tested sources resulted in the production of a bacteriocin with relatively high AMA against *L. sakei* LMGT 2313, the highest activities were obtained when cells were grown in mannitol or lactose. Furthermore, the highest AMA of the produced bacteriocin was attained in the presence of peptone or protease peptone as nitrogen sources (Table 2). In terms of growth conditions, the initial pH of the growth medium showed variable effect on the AMA, since the maximum activity was achieved at pH 7 or 8, while it was abolished at pH 4 or 5. Regarding incubation time, the inhibitory activity was detected after 6 hours of incubation, with a zone of inhibition of 12.33 mm; then it increased gradually with time, showing 13.88 and 18.67 mm zones after incubation for 12 and 18 hours, respectively. In spite of the slight drop in the inhibition zone diameter to 15.33 mm after 24 hours of incubation, the inhibitory activity continued to increase with time, producing a 20.67 mm zone after 30 hours of incubation. When the AMA of avicin A was studied at different incubation temperatures, the inhibitory activity was the highest at 30 °C and 20 °C (corresponding to 18.67- and 16.67- mm zone diameters, respectivley) (Table 3).

**Table 2:** Effect of carbon and nitrogen sources on the production of avicin A, as indicated by the diameter of the zone of inhibition.

Factor	Concentration of the factor (g/100 ml)	Zone of inhibition diameter (mm)*
***Carbon source***:		
Glucose	2	13 ± 0.57
Fructose	2	14.5 ± 0.76
Lactose	2	18.83 ± 0.44^a^
Succrose	2	14.67 ± 0.88
Mannitol	2	21 ± 0.57^b^
***Nitrogen source***:		
Peptone	2.2	21 ± 1^c^
Beef extract	2.2	10 ± 1.15
Yeast extract	2.2	13.83 ± 0.72
Ammonium sulphate	2.2	10.33 ± 1.20
Protease peptone	2.2	16 ± 0.57^d^

*Data representing the average of triplicate values, are shown as mean ± standard error of the mean. ^a^Significantly different from glucose, *p < *0.05. ^b^Significantly different from glucose, fructose, and sucrose, *p < *0.05. ^c^Significantly different from all nitrogen sources, *p < *0.05. ^d^Significantly different from beef extract and ammonium sulphate, *p < *0.05.

**Table 3:** Effect of pH, incubation time, and incubation temperature on the production of avicin A, as indicated by the diameter of the zone of inhibition.

Factor	Zone of inhibition diameter (mm)^*^
***pH***:	
4	0
5	0
6	13.67 ± 0.33
7	19 ± 1^a^
8	21.33 ± 1.33^b^
***Incubation time*** (***hours***):	
6	12.33 ± 0.66
12	13.83 ± 0.44
18	18.67 ± 0.33^c^
24	15.33 ± 1.20
30	20.67 ± 1.20^d^
***Incubation temperature*** (***°C***):	
20	16.67 ± 1.20^e^
25	12 ± 0.57
30	18.67 ± 1.20^f^
37	13.83 ± 0.16
40	8.33 ± 0.88

*Data representing the average of triplicate values, are shown as mean ± standard error of the mean. ^a^Significantly different from pH values (4, 5, 6), *p < *0.05. ^b^Significantly different from pH values (4, 5, 6), *p < *0.05. ^c^Significantly different from incubation time values (6, 12 hours), *p < *0.05. ^d^Significantly different from incubation time values (6, 12, 24 hours), *p < *0.05. ^e^Significantly different from incubation temperature values (25, 40 °C), *p < *0.05. ^f^Significantly different from incubation temperature values (25, 37, 40 °C), *p < *0.05.

## Fractional Factorial Design Analysis

The results of FFD experiments conducted to investigate the effects of media composition and growth conditions on avicin A production are shown in Table 4. Experiments were performed in triplicate, including different combinations of the tested factors and their possible interactions. The application of FFD produced the following regression equation, which is an empirical relationship between bacteriocin activity and test variables in coded units:

$$\begin{array}{rcl}{\rm{Bacteriocin}}\,{\rm{activity}}\,({\rm{Y}}) & = & 174.375+15.625{{\rm{x}}}_{1}\mbox{--}145.625{{\rm{x}}}_{2}+50.625{{\rm{x}}}_{3}\mbox{--}43.125{{\rm{x}}}_{4}\\  &  & \mbox{--}60.625{{\rm{x}}}_{5}\mbox{--}4.375{{\rm{x}}}_{1}{{\rm{x}}}_{2}+24.375{{\rm{x}}}_{1}{{\rm{x}}}_{3}+43.125{{\rm{x}}}_{1}{{\rm{x}}}_{4}75{{\rm{x}}}_{2}{{\rm{x}}}_{3}\\  &  & \mbox{--}14.375{{\rm{x}}}_{1}{{\rm{x}}}_{5}\mbox{--}49.3+36.875{{\rm{x}}}_{2}{{\rm{x}}}_{4}+59.375{{\rm{x}}}_{2}{{\rm{x}}}_{5}\\  &  & +5.625{{\rm{x}}}_{3}{{\rm{x}}}_{4}\mbox{--}56.875{{\rm{x}}}_{3}{{\rm{x}}}_{5}+15.625{{\rm{x}}}_{4}{{\rm{x}}}_{5}\end{array}$$

where *x*_1_ is the carbon source; *x*_2_ is the nitrogen source; *x*_3_ is the initial pH; *x*_4_ is the incubation time; and *x*_5_ is the incubation temperature.

**Table 4:** Effect of the combination of multiple nutrient sources and incubation conditions on avicin A production.

Run No	Nutrient sources		Incubation conditions			Bacteriocin activity (BU/ml)
	Carbon source	Nitrogen source	pH	Time (hours)	Temperature (°C)	
1	Lactose	Protease peptone	7	18	30	320
2	Lactose	Protease peptone	7	30	20	10
3	Lactose	Protease peptone	8	18	20	640
4	Lactose	Protease peptone	8	30	30	80
5	Lactose	Peptone	7	18	20	160
6	Lactose	Peptone	7	30	30	20
7	Lactose	Peptone	8	18	30	640
8	Lactose	Peptone	8	30	20	10
9	Mannitol	Protease peptone	7	18	20	320
10	Mannitol	Protease peptone	7	30	30	40
11	Mannitol	Protease peptone	8	18	30	320
12	Mannitol	Protease peptone	8	30	20	10
13	Mannitol	Peptone	7	18	30	80
14	Mannitol	Peptone	7	30	20	40
15	Mannitol	Peptone	8	18	20	80
16	Mannitol	Peptone	8	30	30	20

Five variables (i.e., mannitol and lactose as carbon sources; peptone and protease peptone as nitrogen sources; pH; incubation time; and incubation temperature) having the highest influence on avicin A production were selected based on preliminary experiments. According to the 2-level 5 variables concept, a 2^5-1^ fractional design was considered, which lead to the 16 runs listed.

**Figure 4:**
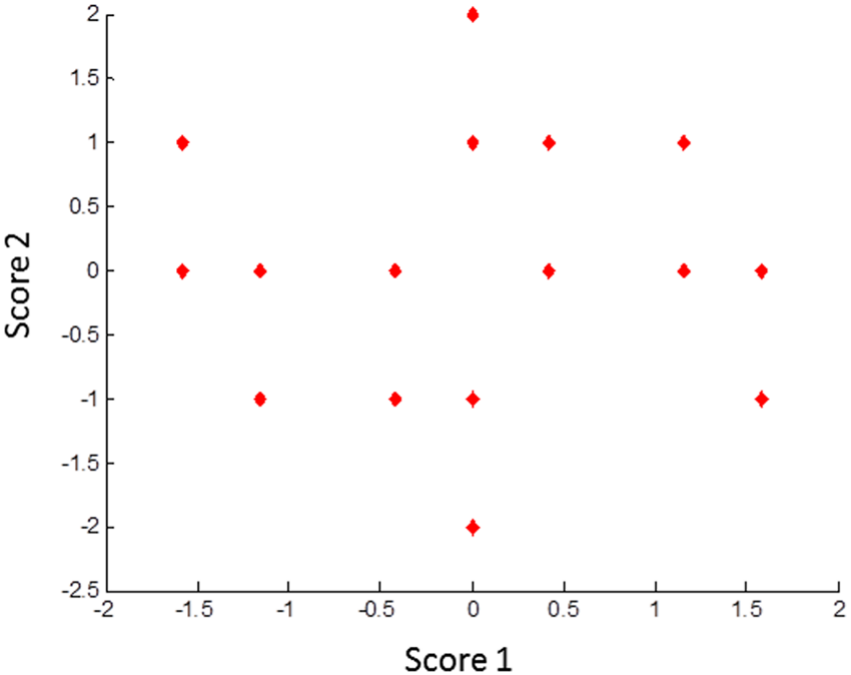
2D scatter plot for the first two principal components after analysis of design matrix, showing the symmetric distribution of test samples in space.

**Figure 5:**
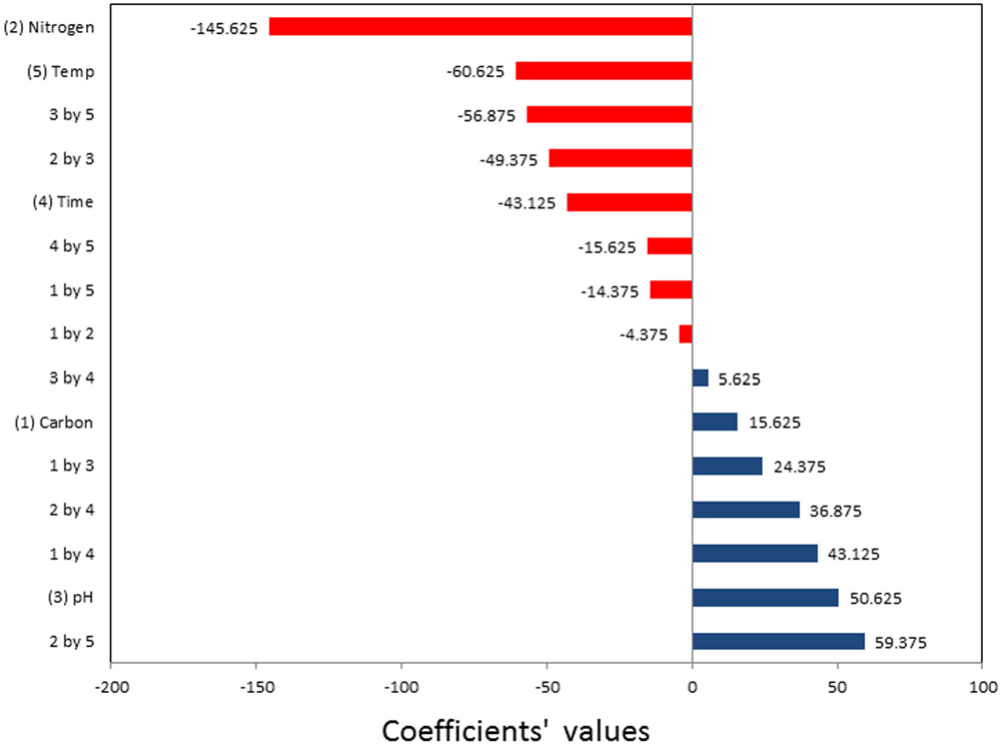
Pareto chart showing the relative magnitude of effects of the five factors identified for FFD and their first-order interactions on avicin A production. Tested factors were: carbon source (1), nitrogen source (2), pH (3), incubation time (4), and incubation temperature (5).

These given values (coefficients) represent the influence of each factor on bacteriocin production and also the impacts of factor-factor interactions. Additionally, the design matrix data were projected onto a two-dimensional (2D) scatter plot (i.e. score plot) to assure the symmetry of the design and the uniform distribution of test samples in space (Fig. 4). The most significant factors were found to be nitrogen source, incubation temperature, pH, and incubation time; among which, only the pH showed a positive effect on bacteriocin production, while all the others had a negative effect (Fig. 5). To explain, raising the initial pH value to 8 enhanced the production of avicin A, while the yield was decreased by increasing incubation temperature and duration of incubation, as well as using peptone as a nitrogen source. Additionally, a number of factor-factor interactions were observed, some of which exerted synergistic effects on bacteriocin production, including the interaction between nitrogen source and incubation temperature, as well as that between carbon source and incubation time (Fig. 5). These positive interactions suggest that bacteriocin production may be optimally achieved either following: (i) 30 h incubation at 30 °C in a medium containing mannitol and peptone; or (ii) 18 h incubation at 20 °C in a medium containing lactose and protease peptone. On the other hand, avicin A production was affected negatively by the interaction between pH and incubation temperature, as well as that between nitrogen source and pH. These negative interactions suggest that bacteriocin yield is expected to decrease either following an incubation: (i) at 30 °C in a medium with a pH of 8; (ii) in a peptone-containing medium adjusted to pH 8; (iii) at 20 °C in a medium with a pH of 7; or (iv) in a medium containing protease peptone that is adjusted to pH 7. Given the results of interaction terms, avicin A production was investigated following 30 h incubation at 30 °C in a medium containing 2 g mannitol and 2.2 g peptone (as carbon and nitrogen sources, respectively) and adjusted to initial pH 7. In our hands, the yield of avicin A under the previously mentioned conditions (2560 BU/ml) was the maximum among all the conditions we examined.

## Physicochemical Characterizations of LDHs, Avicin A, and Avicin A-LDH Nanocomposites

**Figure 6:**
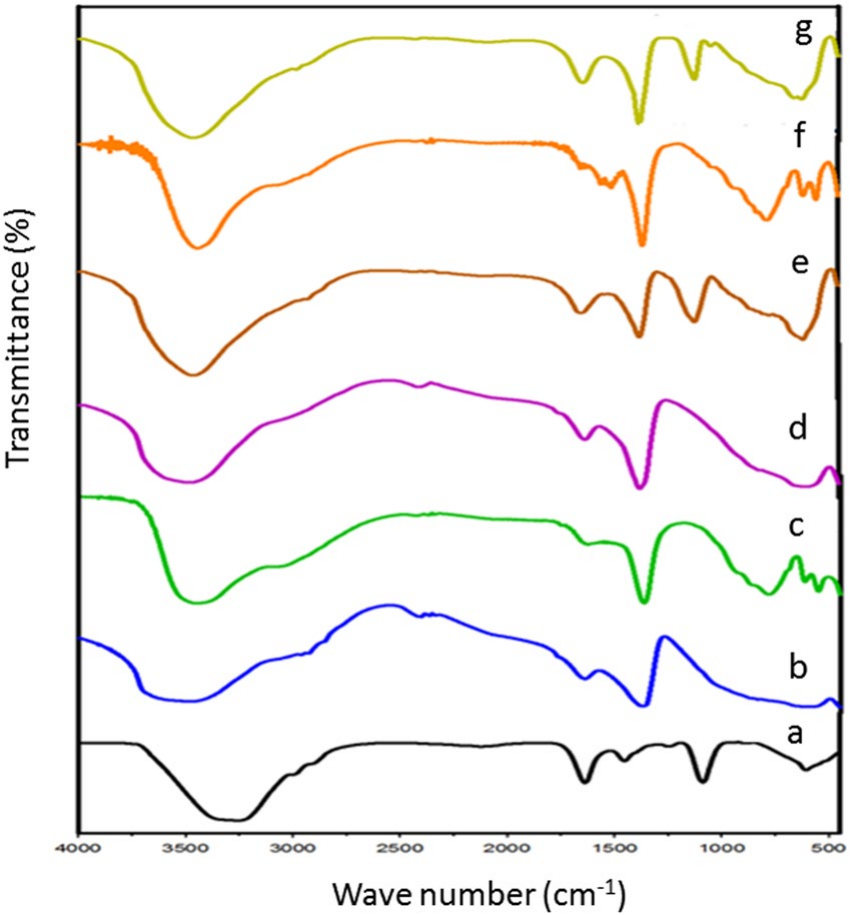
FT-IR spectra of (**a**) avicin A, (**b**) MgAl-CO_3_ LDH, (**c**) ZnAl-CO_3_ LDH, (**d**) MgAl-NO_3_ LDH, (**e**) avicin A-MgAl-CO_3_ LDH nanocomposite, (**f**) avicin A-ZnAl-CO_3_ LDH nanocomposite, and (**g**) avicin A-MgAl-NO_3_ LDH nanocomposite.

As shown in Fig. 6(b–d), the FT-IR spectra revealed an intense absorption band at around 3500 cm^−1^ for the three LDHs, which can be attributed to the OH stretching of hydroxyl group and water molecules adsorbed on LDHs. The spectra also exhibited a strong absorption band around 1365 cm^−1^, mainly due to the presence of carbonate group. Regarding the nitrate group, the characteristic band caused by the stretching of the nitrate anions was detected at 1381 cm^−1^. In the FT-IR spectra of avicin A (Fig. 6a), there were four absorption bands (at 3256, 1636, 1087, and 606 cm^−1^) due to unknown functional groups of this bacteriocin. After the formation of nanocomposites with LDHs, all the bands corresponding to avicin A functional groups were still clearly visible in avicin A-MgAl-CO_3_ LDH and avicin A-MgAl-NO_3_ LDH nanocomposites (Fig. 6e and g). In the FT-IR spectra of avicin A-ZnAl-CO_3_ LDH nanocomposite, the two peaks of avicin A at 3256 and 606 cm^−1^ were overlapped with peaks of LDH and shifted to 3446 and 611 cm^−1^ (Fig. 6f).

**Figure 7:**
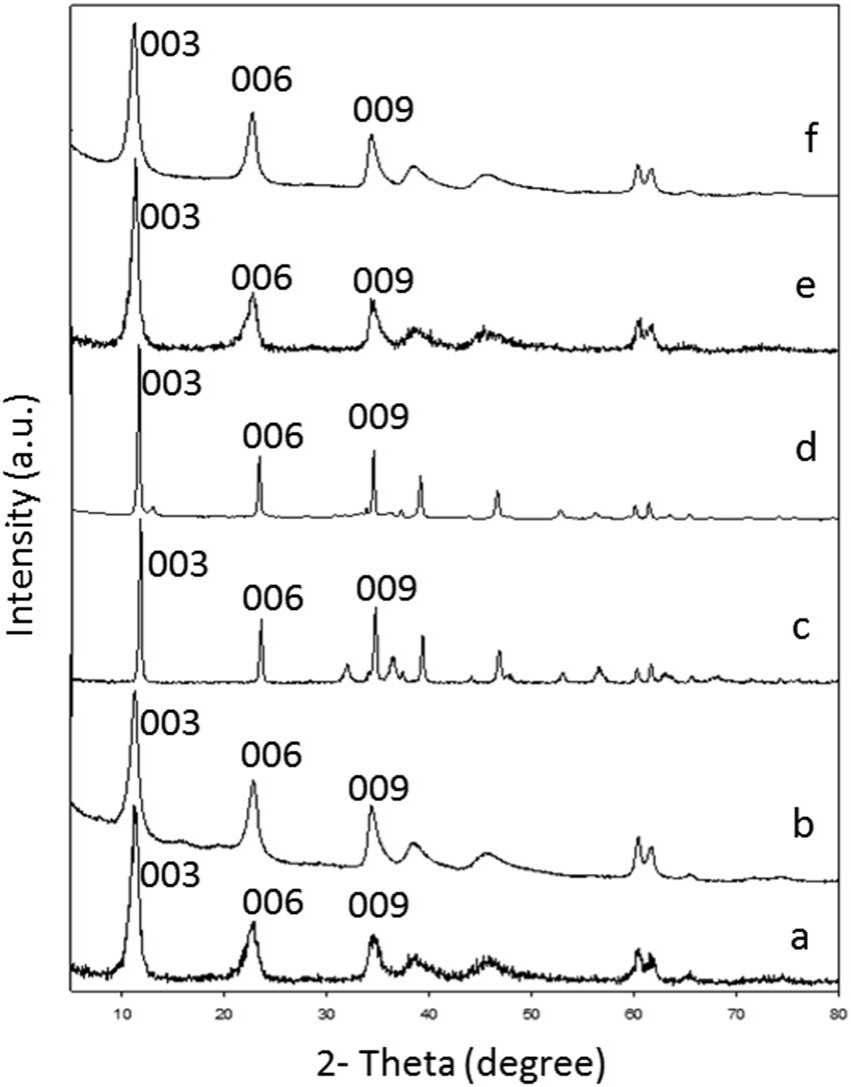
XRD patterns of (**a**) MgAl-CO_3_ LDH, (**b**) avicin A-MgAl-CO_3_ LDH nanocomposite, (**c**) ZnAl-CO_3_ LDH, (**d**) avicin A-ZnAl-CO_3_ LDH nanocomposite, (**e**) MgAl-NO_3_ LDH, and (**f**) avicin A-MgAl-NO_3_ LDH nanocomposite.

As shown in the XRD analysis presented in Fig. 7, all the LDHs examined (MgAl-CO_3_, ZnAl-CO_3_, and MgAl-NO_3_ LDHs) as well as the resulting avicin A-LDH nanocomposites exhibited sharp and symmetrical well-ordered reflection peaks, such as (003), (006), (009). For MgAl-CO_3_ LDH (Fig. 7a), the peak corresponding to the reflection intensity (100%) at 2θ = 11.2905 indicated that the basal spacing was 7.83724 [Å] and the crystallite size was 13.1 nm. For ZnAl-CO_3_ LDH (Fig. 7c), the peak corresponding to the reflection intensity (100%) at 2θ = 11.7271 indicated that the basal spacing was 7.54638 [Å] and the crystallite size was 48.5 nm. Also, the XRD analysis of ZnAl-CO_3_ LDH showed an additional peak centred at 2θ = 34.7, and is thought to be most probably due to traces of zinc oxide phase (Fig. 7c). In the case of MgAl-NO_3_ LDH (Fig. 7e), the peak corresponding to the reflection intensity (100%) at 2θ = 11.3158 indicated that the basal spacing was 7.81972 [Å] and the crystallite size was 37.9 nm. Regarding the XRD patterns of avicin A-LDH nanocomposites (Fig. 7b,d and f), there was a similarity between these samples and the free LDHs in the d-space and position of all peaks with a slight decrease in intensity of peaks.

**Figure 8:**
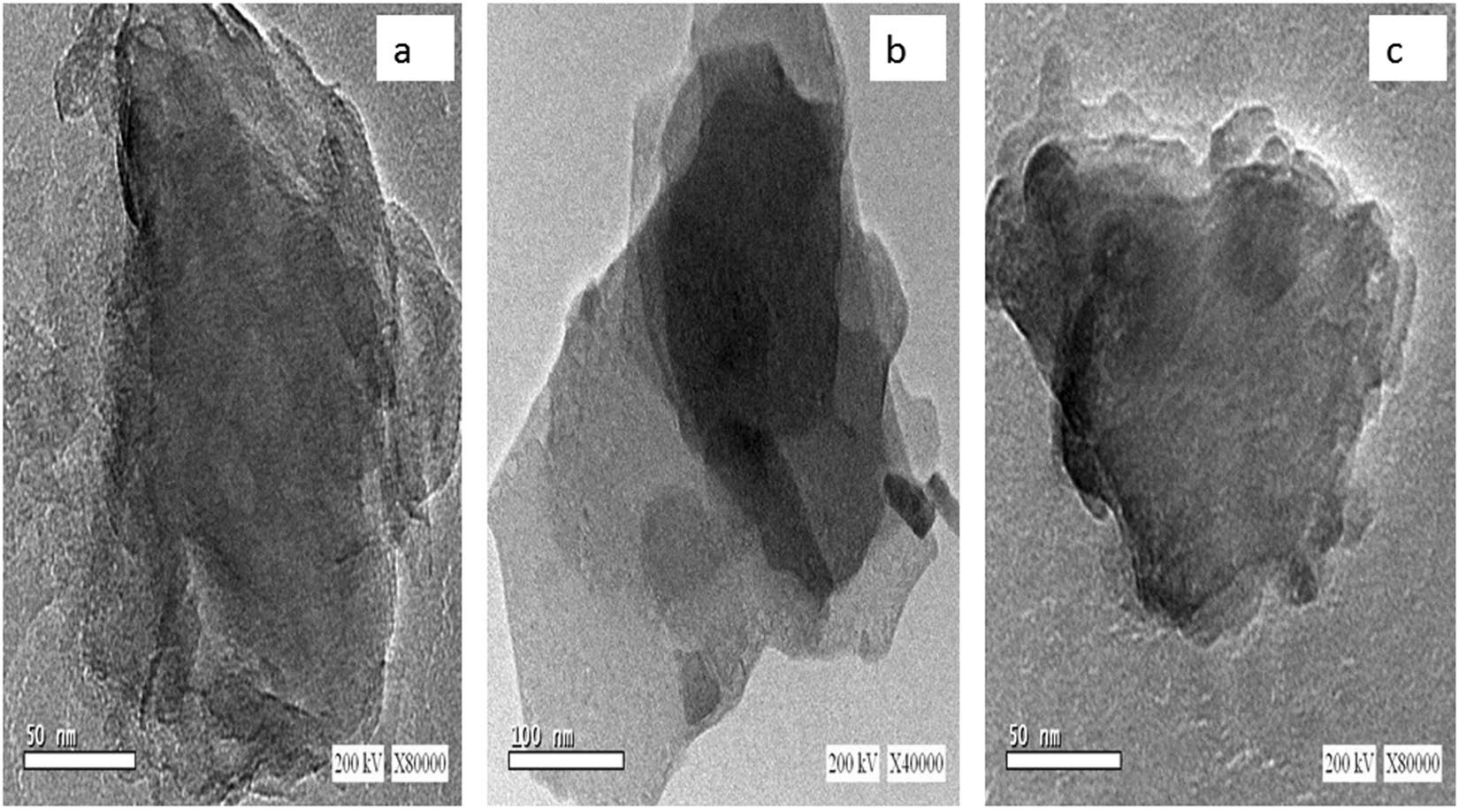
TEM images of (**a**) MgAl-CO_3_ LDH, (**b**) ZnAl-CO_3_ LDH, (**c**) MgAl-NO_3_ LDH.

The morphology of LDHs was also examined using TEM, which revealed thin nanosheets arranged in layers and stacked upon each other with a particle size of 50 nm for MgAl-CO_3_ LDH and MgAl-NO_3_ LDH (Fig. 8a and c), while it was 100 nm for ZnAl-CO_3_ LDH (Fig. 8b).

## The Antibacterial Activity of Avicin A-LDH Nanocomposites

**Figure 9:**
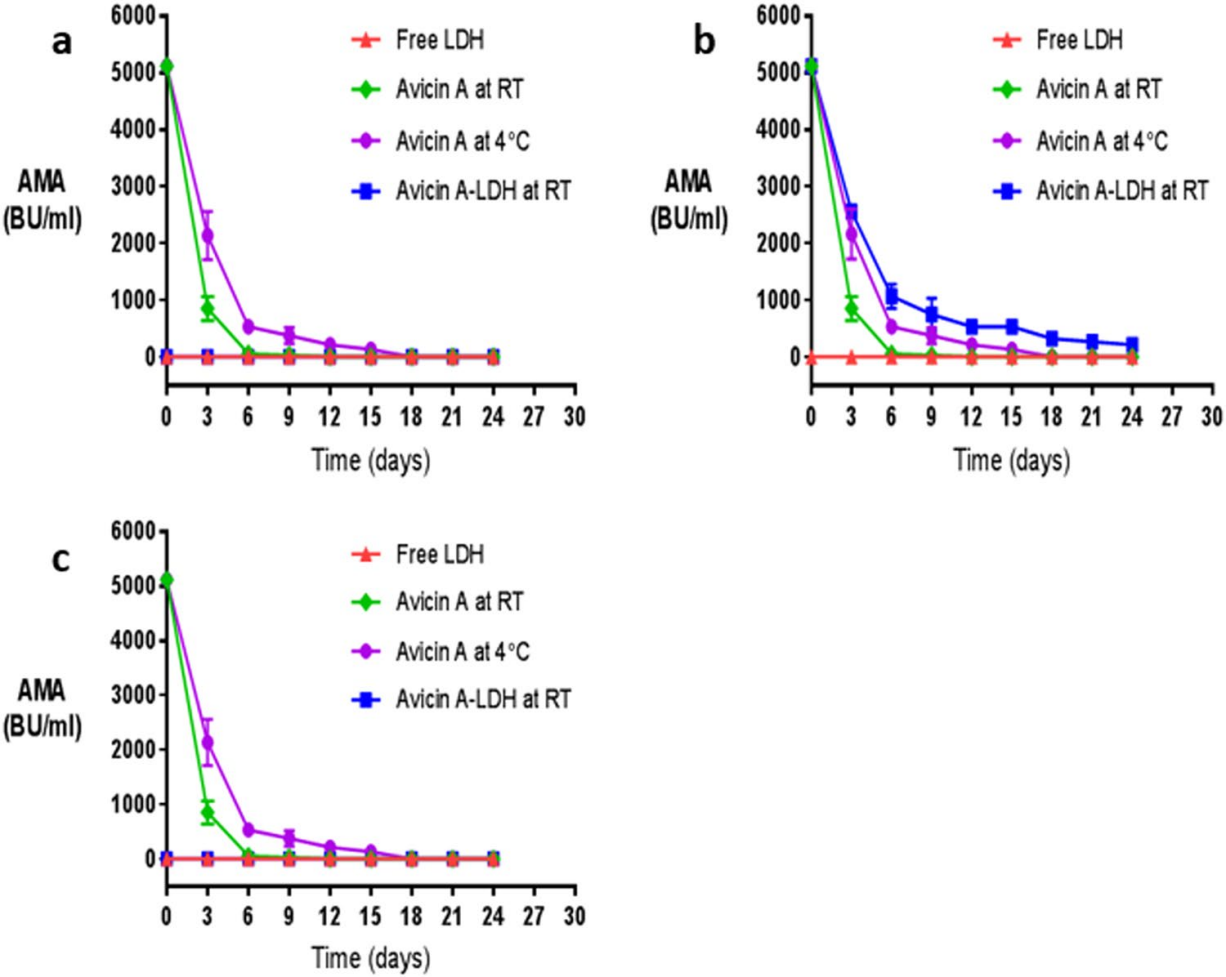
The AMA of the nancocomposites of (**a**) avicin A-MgAl-CO_3_ LDH, (**b**) avicin A-ZnAl-CO_3_ LDH, and (**c**) avicin A-MgAl-NO_3_ LDH against *L. sakei* LMGT 2313 as a function of storage time. The AMA (expressed as BU/ml) was monitored as a function of storage time for up to 24 days. Each point represents the mean and standard error of triplicate determinations. In each graph, the AMA of the resulting avicin A-LDH nanocomposites (■) is shown in comparison with the activity of the free LDHs (▲), the RT-stored avicin A (◆), and the 4 °C-stored avicin A (●). In graphs a and c, please note that the line representing the AMA of the free LDHs is superimposed on that of the resulting avicin A-LDH nanocomposites.

The stability and spectrum of AMA of the avicin A-LDH nanocomposites were compared with those obtained from the free avicin A and the different LDH precursors. In these assays, a free avicin A sample with a concentration of 249 mg/ml and AMA of 40960 BU/ml (Table 5) was used to form the various avicin A-LDH nanocomposites, which all showed an entrapment efficiency of over 50%. To evaluate the bacteriocin stability, the AMA of the previously mentioned preparations were tested against *Lactobacillus sakei* LMGT 2313 at 72 h intervals for up to 24 days (Fig. 9).

**Table 5:** Steps of avicin A purification.

Purification step	Volume (ml)	Recovery (%)	Protein concentration^*^ (mg/ml)	AMA (BU/ml)
Culture supernatant	250	100	17	1280
Ammonium sulphate precipitate	10	128	249	40960

*The protein concentration was determined using the Total Protein Kit (Greiner Diagnostic GmbH).

Among all the nanocomposites obtained in this study, only the ZnAl-CO_3_ LDH showed a synergistic effect with avicin A when stored at RT (Fig. 9b). Even when the avicin A-ZnAl-CO_3_ LDH nanocomposite was stored at RT for 24 days, it retained about 6.14% of its initial AMA (320 out of 5120 BU/ml); whereas the activity of the free avicin A that has been stored at RT and at 4 °C declined nearly to baseline levels after 6 and 15 days of incubation, respectively, (Fig. 9b). On the other hand, both MgAl-CO_3_ and MgAl-NO_3_ LDHs did not reveal any synergistic effect with the free avicin A, and the AMA of the latter was even abolished after nanocomposite formation with both LDH precursors (Fig. 9a and c).

Furthermore, the AMA of all these nanocomposite preparations was tested against a group of Gram-negative and -positive bacteria that are normally resistant to the free Avicin A, including *E. coli*, MRSA, and MSSA. Unfortunately, the avicin A-LDH nanocomposites did not show any remarkable AMA against the bacterial strains investigated, suggesting that the conjugation approach presented here serves mainly as a means of stably maintaining the activity of this bioactive molecule for an extended period of time.

Avicin A is a subclass IIa bacteriocin produced by *E. avium* HF86 that exhibits a potent AMA against many species of Gram-positive bacteria, including the food-borne pathogen *L. monocytogenes* ^17^. It is well accepted that bacteriocin production can be considerably affected by media composition and growth conditions^18, 19^. In this study, we investigated the effect of different carbon sources, nitrogen sources, pH, incubation time, and incubation temperature on the production of avicin A.

In our preliminary screening, almost all the tested factors had an impact on the AMA of avicin A, but with varying magnitude. Regarding carbon and nitrogen sources, a study conducted by Todorov *et al*.^20^ has reported the effect of different concentrations of carbon and nitrogen sources on the production of plantaricin ST31 from *Lactobacillus plantarum* ST31, in which lactose, sucrose, glucose, beef extract, and yeast extract have been shown to be the most influential factors affecting the activity of the bacteriocin produced. These results agree in part with the data obtained in our study, where mannitol, lactose, peptone, and protease peptone were the most effective carbon and nitrogen sources. In agreement with its documented influence on bacteriocins production^21^, pH values above 6 demonstrated a considerable effect on the AMA of avicin A. This finding is interesting and needs further investigation, because it contradicts with the posttranslation processing of prepediocin to the active bacteriocin, pediocin AcH, which has been previously shown to occur at a pH below 5^22^. We can speculate that this processing may be confined to pediocin AcH only, and does not extend to include avicin A. In our hands, the activity of avicin A increased gradually with increasing the incubation time and reached the highest level at 30 h, which may be attributed to the accumulation of bacteriocin during the exponential phase^23, 24^. Similar pattern has been reported for the bacteriocin produced from *Lactobacillus murinus* AU06, which showed the highest activity in the period between 18 h and 35 h^10^. In accordance with previous studies^25, 26^, the optimal production of avicin A occured at 30 °C. However, several other reports have highlighted that the optimum production of bacteriocin can be obtained at different incubation temperatures^21, 27^, which was not the case in avicin A. This discrepancy could be attributed to the variations among lactic acid bacteria regarding the ideal incubation temperature required for production of bacteriocins.

In our study, the fractional factorial experimental design was chosen not only to limit the number of runs required, but also to identify factor-factor interactions. The first step to increase the yield is to identify the factors that are of a significant influence on the desired response. The obtained results showed that nitrogen source, incubation temperature, pH, and incubation time strongly affect avicin A production. Based on the interaction terms obtained from the FFD experiments, the maximum avicin A production was achieved in presence of mannitol and peptone at 30 °C, with pH adjustment at 7, and incubation time 30 h. Our findings are generally in agreement with previous investigations, which have demonstrated the strong influence of media composition, pH, incubation time, and incubation temperature on the production of bacteriocins^28, 29^.

Direct application of bacteriocins in food as biopreservative is faced by some challenges that limit the efficiency of these molecules, including the loss of their AMA due to proteolytic degradation and/or unfavorable interactions with other food components^4^. Herein, different LDHs were used to form nanocomposites with avicin A, in an attempt to improve the stability and activity of the latter. To the best of our knowledge, this is one of the very few studies to investigate the impacts of LDHs on the AMA of bacteriocins. Characterization of LDHs, avicin A, and avicin A-LDH nanocomposites by XRD and FT-IR revealed the successful preparation of these nanocomposites. When the free LDHs and the avicin A-LDH nanocomposites were subjected to XRD analysis, they all showed the same peaks and almost the same d-space, indicating that avicin A was not intercalated into the interlayers of the LDHs. Also, unfavourable electrostatic interactions between the positively charged avicin A and the net positive charge at the surface of LDH^30–32^ could also eliminate the possibility of having adsorption or entrappment as the main mechanism behind this interaction. Therfore, we can assume that the avicin A-LDH nanocomposites produced in this study have been made through functional group conjugation rather than simple complexation. The successful formation of the avicin A-LDH nanocomposites was further confirmed by FT-IR spectra, where all peaks corresponding to the functional groups of LDHs and avicin A were clearly observed. The overlapping and shifting that happened in the FT-IR of avicin A-ZnAl-CO_3_ LDH nanocomposite could be attributed to the strong conjugation effect between functional groups in the bacteriocin and the ZnAl-CO_3_ LDH^33^. These results are in accordance with those obtained regarding the intercalation of isoniazid into MgAl LDH, where some peaks of the isoniazid have been shown to be overlapped with peaks of LDH and shifted^33^.

To investigate the effect of prolonged storage on the various avicin A-LDH nanocomposites, their antimicrobial activities were tested against *L. sakei* LMGT 2313 for up to 24 days. Surprisingly, the avicin A-ZnAl-CO_3_ LDH nanocomposite possessed markedly improved stability and activity compared to the RT-stored and the 4 °C-stored avicin A. This preliminary result seem more promising than those obtained by other nanodelivery systems, such as nanoliposomal encapsulation, which in certain cases, as with the AMA of the bacteriocin P34 when tested against *L. monocytogenes* ATCC 7644^34^, have resulted in a decrease of the biological activity.

Regarding the possible mode of action of avicin A-ZnAl-CO_3_ LDH nanocomposite, it seems reasonable to suggest that it is a result of two steps, the first of which is the immobilization of the negatively charged bacteria by adsorption to the LDH; while the second step is attributed to the bactericidal effect of avicin A. This is in agreement with a study conducted by Yang and his colleagues^16^, in which lysozyme-LDH has been shown to exhibit much higher antibacterial activity against *S. aureus* than the free lysozyme. On the other hand, avicin A-MgAl-CO_3_ and avicin A-MgAl-NO_3_ LDH nanocomposites failed to demonstrate remarkable AMA against the indicator strain. Although it may be claimed that avicin A could not be released from MgAl-CO_3_ and MgAl-NO_3_ LDHs to provide its AMA, but further research is required to uncover the exact mechanism of this failure.

In conclusion, the present study revealed the influential impact of nitrogen sources, incubation temperature, pH, and incubation time on avicin A production. In our hands, the maximum yield of avicin A (2560 BU/ml) was achievable following 30 h incubation at 30 °C in a medium containing mannitol and peptone and adjusted to initial pH 7. The avicin A-ZnAl-CO_3_ LDH nanocomposite demonstrated a remarkable AMA lasting for at least 24 days, whereas the activity of the free avicin A lasted only for 6 and 15 days, following storage at RT and at 4 °C, respectively. In this sense, it is suggested that the approach used in this study may have a true potential for improving the applicability of bacteriocins in the food and biomedical industries. Further studies are under consideration to examine this possibility, as well as to gain a better understanding of the properties and the exact formation mechanism of the nanocomposite used in this work.

## Chemicals and Bacterial Strains

All chemicals, salts, as well as carbon and nitrogen sources, were obtained from Difco Laboratories (Detroit, USA). *E. avium* HF86, the producer strain of avicin A, was kindly provided by one of the authors (A. O. El-Gendy), and its purity was checked on bile esculin agar (Laboratory Conda, Spain). The identity of this bacteriocinogenic strain was confirmed by sequencing of its 16 S rRNA gene, followed by BLAST homology search at the NCBI database. The *in vitro* susceptibility of this isolate against different antibiotics was also investigated. Unless otherwise noted, cultures of this strain were propagated in Man Rogosa Sharpe (MRS) broth (Oxoid, England) at 30 °C. The stock cultures of all bacterial strains used in this study, including the sensitive indicator strain *Lactobacillus sakei* LMGT 2313, were kept frozen at −80 °C in MRS/glycerol broth (60:40 v/v) until analysed.

## Production and Partial Purification of Avicin A

An overnight culture of *E. avium* HF86 was inoculated at 1% into 100 ml MRS broth and incubated at 30 °C for 18 h in a static condition. The bacterial cells were removed by centrifugation at 10,000 rpm for 20 min, at 4 °C. The cell-free supernatant was adjusted to pH 6.5 by the addition of 1 N NaOH and used as a crude bacteriocin preparation to study the effect of growth conditions and media composition on bacteriocin production.

To form nanocomposites with LDH, 500 ml of MRS broth were inoculated with 1% (v/v) of an overnight culture of the producer strain. The bacteriocin was partially purified from the cell-free supernatant by precipitation with 50% (w/v) ammonium sulfate, followed by centrifugation at 10,000 rpm for 30 min, at 4 °C. The precipitated protein was dissolved in 10 ml distilled water, and therefore, the finally obtained protein was 50x concentrated. The total amount of protein was determined using the Total Protein Kit (Greiner Diagnostic GmbH), and the concentrated bacteriocin was used for conjugation with LDH nanoparticles.

## Investigation of the Activity and Identity of Avicin A

The antimicrobial activity (AMA) of avicin A was evaluated by the agar well-diffusion method, using the previously mentioned indicator strain. In addition to determination of inhibition zone diameter, bacteriocin samples were serially diluted two-fold, and the reciprocal of the highest inhibitory dilution was used to express the arbitrary activity units (AU) per millilitre. To verify the protein nature of avicin A, the culture supernatant was treated with 1 mg/ml of proteinase K enzyme (Sigma) for 60 min at 37 °C. The expression of the gene encoding the avicin A was also confirmed by subjecting the bacterial mRNA to reverse transcription PCR (RT-PCR), then the resulting cDNA was PCR amplified using specific primers (Table 1), and the amplified products were visualized by electrophoresis in 2% agarose gel stained with ethidium bromide to verify band size.

## Effect of Media Composition and Growth Conditions on Avicin A Production

Screening of the effects of media composition and growth conditions on avicin A production were conducted using 100 ml of MRS broth inoculated with 1% (v/v) of an overnight culture of *E. avium* HF86 and subjected to different parameters, such as carbon and nitrogen source supplementation (Table 2), various pH values (4, 5, 6, 7 and 8), incubation time (6, 12, 18, 24, and 30 h), and incubation temperatures (20, 25, 30, 37, and 40 °C) (Table 3). Following incubation, samples of the produced bacteriocin were collected to examine the AMA by determination of inhibition zone diameter.

## The Fractional Factorial Experimental Design (FFD)

Based on the preliminary screening of media composition and growth conditions carried out by one-variable-at-a-time (OVAT) approach, the most important five factors were examined, with two levels each, for their combined effect on avicin A production (Table 4). FFD (2^k−1^) was used to run 16 independent experiments^13^. For five factors, the model’s equation is:

$$\begin{array}{rcl}{\rm{Y}} & = & {b}_{0}+{b}_{1}{x}_{1}+{b}_{2}{x}_{2}+{b}_{3}{x}_{3}+{b}_{4}{x}_{4}+{b}_{5}{x}_{5}+{b}_{12}{x}_{1}{x}_{2}+{b}_{13}{x}_{1}{x}_{3}+{b}_{14}{x}_{1}{x}_{4}+{b}_{15}{x}_{1}{x}_{5}\\  &  & +{b}_{23}{x}_{2}{x}_{5}+{b}_{24}{x}_{1}{x}_{5}+{b}_{25}{x}_{1}{x}_{5}+{b}_{34}{x}_{1}{x}_{5}+{b}_{35}{x}_{1}{x}_{5}+{b}_{45}{x}_{1}{x}_{5}\end{array}$$

where, Y is the predicted response (bacteriocin activity in terms of BU/ml); *b*_0_ the intercept; *b*_1_, *b*_2_, *b*_3_, *b*_4_, and *b*_5_ are the linear coefficients which express the direct effect of factors on response; *b*_12_, *b*_13_, *b*_14_, *b*_15_, *b*_23_, *b*_24_, *b*_25_, *b*_34_, *b*_35_, and *b*_45_ are interaction coefficients reflecting trends and magnitude of interaction among the represented factors; *x*_1_ is the carbon source; *x*_2_ is the nitrogen source; *x*_3_ is the initial pH; *x*_4_ is the incubation time; and *x*_5_ is the incubation temperature.

## Preparation of LDH Nanoparticles

MgAl-CO_3_, ZnAl-CO_3_, and MgAl-NO_3_ LDHs were synthesized by the co-precipitation method previously described by Mohanambe and colleague^35^, and the molar ratio of divalent cations to trivalent cations (M^2+^:M^3+^) was kept at 3:1 in all cases. Briefly, an aqueous solution containing NaOH (0.5 M) was added drop wise into a solution containing the specific amounts of the divalent and trivalent cations, the final pH of the solution was maintained between 8 and 10, and the resulting slurry was aged at 65 °C for 18 h while stirring. For preparing MgAl-NO_3_ LDH, the experiment was done carefully under atmospheric nitrogen. Finally, the resulting precipitate was washed several times with hot deionized water and allowed to dry overnight at 60 °C.

## Formation of Avicin A-LDH Nanocomposites

Nanocomposites were prepared by adding the partially purified avicin A to the LDH nanoparticles in a molar ratio of 2:1, and the mixture was allowed to age at ≤4 °C for 72 h, with vigorous stirring under nitrogen. The slurry was subjected to centrifugation (3,400 rpm) for 15 min at 4 °C. The resulting precipitate was washed thrice with deionized water, centrifuged at 3,400 rpm, and dried at room temperature (RT). The concentration and the antimicrobial activity of the residual non-conjugated avicin A, present in the supernatant fraction obtained after the first centrifugation step, were measured to determine the entrapment efficiency.

### Physicochemical Characterizations of LDHs, Avicin A, and Avicin A-LDH Nanocomposites

In this study, avicin A was characterized by Fourier transform infrared (FT-IR) spectroscopy, while the LDH nanoparticles and avicin A-LDH nanocomposites were characterized by X-ray diffraction (XRD) and FT-IR spectroscopy. XRD patterns were recorded by PANalytical Empyrean Diffractometer system using Cu (Kα) radiation (λ = 1.54 Å) at 40 kV and 30 mA, with 2θ angles ranging from 5° to 79.9°. FT-IR spectra were recorded using a Vertex-70 spectrophotometer (Bruker, Germany) in the 4000 − 400 cm^−1^ range using the KBr pellet technique. To study the morphology of LDH nanoparticles, the synthesized LDHs were examined using Transmission Electron microscope (TEM) (JEOL-JEM 2100, Japan) with an acceleration voltage of 200 KV.

## Stability and Spectrum of Activity of the Avicin A-LDH Nanocomposites in Comparison with the Free Materials

To evaluate the stability of their antimicrobial activity after prolonged storage, the AMA of RT-stored avicin A, 4 °C-stored avicin A, the three nanocomposites (avicin A-MgAl-CO_3_ LDH, avicin A-ZnAl-CO_3_ LDH, and avicin A-MgAl-NO_3_ LDH), and the free LDH nanoparticles, all stored at RT, were tested against *Lactobacillus sakei* LMGT 2313 at 72 h intervals for up to 24 days. Furthermore, the AMA of all these preparations was tested against bacterial strains that are normally resistant to the free Avicin A, including *Escherichia coli* ATCC 25922, methicillin-resistant *Staphylococcus aureus* (MRSA; ATCC 43300), and methicillin-sensitive *Staphylococcus aureus* (MSSA; ATTC 25923).

## Statistical Analysis

All experiments were carried out in triplicate and in a randomized order. Results were analysed by statistical software (Matlab; version 7.1.0.246 (R14); Math Works, Inc., Natick, MA) to calculate the coefficients, and hence, evaluate the effects of linear and interaction terms of independent variables.

## Electronic supplementary material

Supplementary Information
