# Supplementary material for: Enhancement of the productivity of the potent bacteriocin avicin A and improvement of its stability using nanotechnology approaches
Source: Sci Rep. 2017 Sep 6;7:10604. doi: 10.1038/s41598-017-10157-9 (PMC5587769; doi:10.1038/s41598-017-10157-9)
Supplement: Supplementary file 1 — Supplementary Information [file 41598_2017_10157_MOESM1_ESM.docx]

# Supplementary Information

# Journal: Scientific Reports

**Manuscript number:** SREP-17-20780A

**Revised manuscript Title:** Enhancement of the productivity of the potent bacteriocin avicin A and improvement of its stability using nanotechnology approaches

**Authors:** Hazem A. Fahim, Waleed M. A. El Rouby, Ahmed O. El-Gendy, Ahmed S. Khairalla, Ibrahim A. Naguib, and Ahmed A. Farghali

**Corresponding authors:** Ahmed O. El-Gendy and Ahmed S. Khairalla


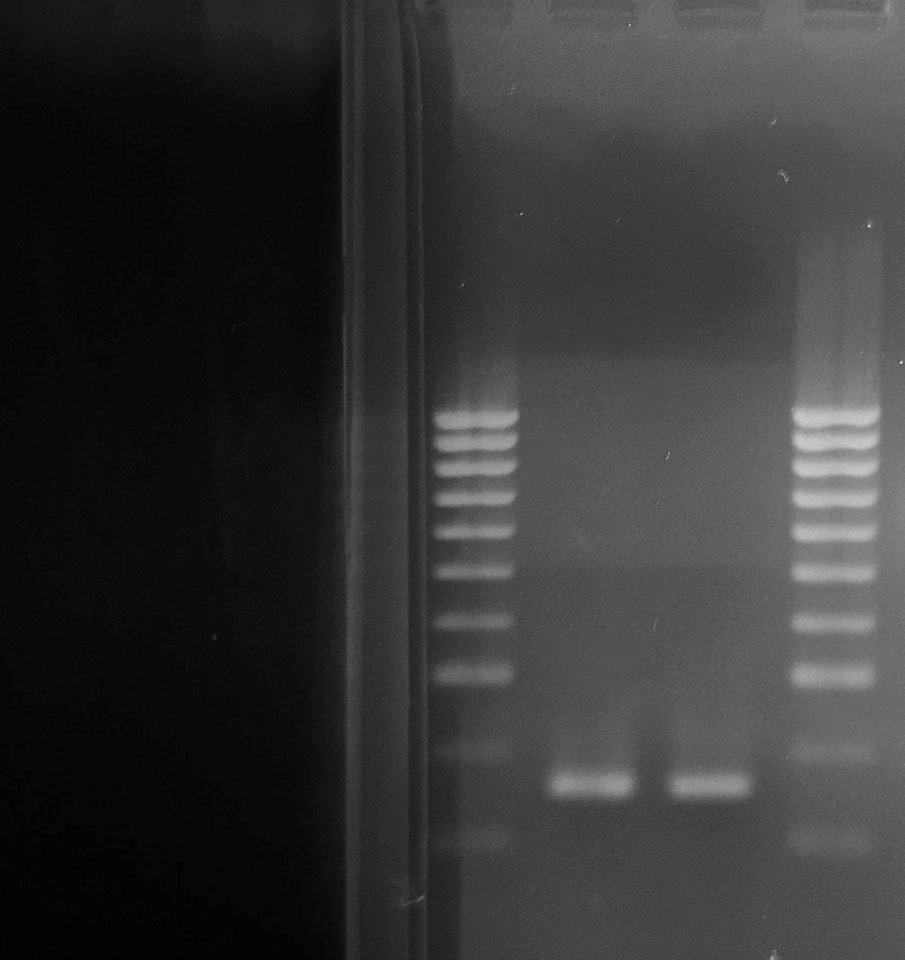


**Fig. S1. RT-PCR detection of avicin A mRNA.** Total RNA (1 μg) isolated from *E. avium* HF86 cells was reverse transcribed, and the resulting cDNA was amplified by a 35-cycle PCR with the primer pairs AV-F and AV-R (Table 1). The PCR product was visualized after agarose gel electrophoresis (2%) with ethidium bromide. The middle two lanes (lanes 2 and 3) show the reverse‑transcribed PCR product of avicin A with the expected product size (156 bp). The DNA molecular size marker is shown in the leftmost and the rightmost lanes (lanes 1 and 4).
